# Artificial Intelligence and Machine Learning in Sexual Health and Dysfunction Across the Cancer Care Continuum: A Systematic Review

**DOI:** 10.3390/cancers17183025

**Published:** 2025-09-16

**Authors:** Vivian Salama, Brandon M. Godinich, Peyton M. Lilly, Phillip M. Pifer, Adrienne L. Duckworth, Samantha J. Hall, Maher Alabboodi, R. Alfredo Siochi, David A. Clump, Ashkan Emadi

**Affiliations:** 1 Department of Medical Oncology, WVU School of Medicine, West Virginia University Cancer Institute, West Virginia University, Morgantown, WV 26506, USA; aduckwor@hsc.wvu.edu (A.L.D.); shall19@hsc.wvu.edu (S.J.H.); ashkan.emadi@hsc.wvu.edu (A.E.); 2 Department of Radiation Oncology, School of Medicine, West Virginia University Cancer Institute, West Virginia University, Morgantown, WV 26506, USA; ppifer@hsc.wvu.edu (P.M.P.); mjalabboodi@hsc.wvu.edu (M.A.); rasiochi@hsc.wvu.edu (R.A.S.); dclump@hsc.wvu.edu (D.A.C.); 3 Department of Medical Education, Paul L. Foster School of Medicine, Texas Tech Health Sciences Center, El Paso, TX 79430, USA; brandon.m.godinich@uth.tmc.edu; 4 Department of Medical Education, WVU School of Medicine, West Virginia University Health Sciences Center, Morgantown, WV 26506, USA; pl00020@mix.wvu.edu

**Keywords:** artificial intelligence, machine learning, sexual health, sexual dysfunction, cancer, oncology

## Abstract

*Background:* Sexual dysfunction is a common, under-addressed consequence of cancer, impacting patient quality of life. Artificial intelligence (AI) and machine learning (ML) are emerging tools in oncology with potential to improve prediction, assessment, and management of sexual health outcomes. The main aim of the review was to investigate the current application of AI-ML models in sexual health across the cancer care continuum. *Methods:* A systematic, PROSPERO-registered review followed PRISMA guidelines. PubMed, EMBASE, and Web of Science were searched for English-language original human studies published up to 18 February 2025, using the terms: “artificial intelligence,” “machine learning,” “sexual health,” etc. Only studies in cancers were included. Two reviewers screened studies applying AI-ML to sexual health in cancer care. Extracted data included study characteristics, AI-ML method, and model performance. Risk of bias was assessed using PROBAST, and adherence to reporting standards was evaluated using TRIPOD+AI. *Results:* Twenty-eight studies (2002–2025) addressed AI in sexual health across five cancer care phases: prevention, early detection/screening, diagnosis, treatment, and survivorship. Natural language processing (NLP) models were used for automated data extraction, and DL was used for imaging analysis. Most studies applied regression models (*n* = 18), gradient boosting models (*n* = 14), and neural networks (*n* = 14). Random Forest achieved the highest performance [median AUC 0.98 (range 0.91–0.99), sensitivity 0.98, specificity 0.99, and F1 score 0.98]. Lack of calibration assessment was detected (*n* = 6, 21.4%). Overall adherence to TRIPOD + AI guidelines was 60% with high risk of bias (100%). *Conclusions:* Artificial intelligence offers promising avenues for sexual health prediction, follow-up, and management across cancer care continuum phases. However, methodological limitations and lack of standardized outcome measures highlight the need for higher quality studies and external validation to support clinical integration.

## Simple Summary

Many people with cancer experience sexual health problems as a result of their disease or treatment, but this important aspect of quality of life is often overlooked in research and clinical care. New technologies such as artificial intelligence and machine learning can help clinicians and researchers better understand, predict, and manage these problems by analyzing large amounts of health data. In this study, we reviewed all published research that applied these advanced technologies to sexual health across different stages of cancer care, from prevention and diagnosis to treatment and survivorship. We found that while some early results show potential, most studies have important limitations, such as lack of standard measures, reporting transparency, and proper performance reporting. Our findings highlight the need for more rigorous and high-quality research in digital technologies in cancer. With further development, these tools could support more personalized care and improve sexual health outcomes for cancer survivors.

## 1. Introduction

Sexual dysfunction (SD) and sexual health problems are common distressing and often under-addressed issues among both male and female patients with cancer and without cancer [1,2]. Sexual problems not only impact patient outcomes but also significantly affect their quality of life (QoL) [3,4]. These sexual challenges can arise during diagnosis, treatment, or post-treatment survivorship [1,2,4,5].

**Figure 1:**
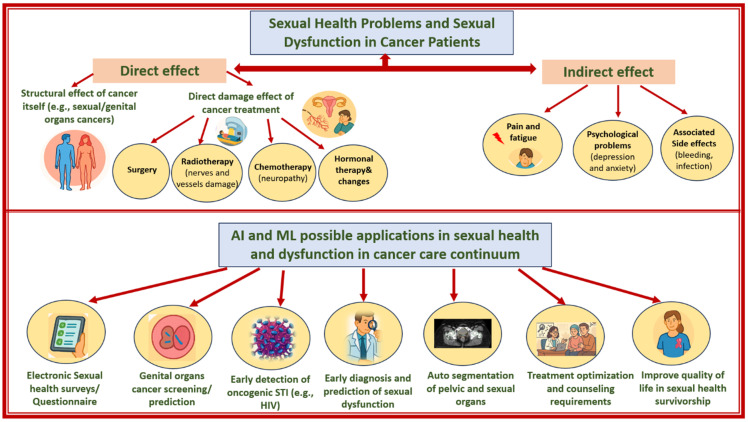
Summary of causes of sexual health problems and dysfunction in patients with cancers (UPPER) and the possible applications of artificial intelligence and machine learning in sexual health and dysfunction in the cancer care continuum (LOWER).

Sexual problems in patients with cancer are multifactorial, arising from both direct and indirect effects. Cancers involving reproductive organs (e.g., cervical or prostate) and treatments such as surgery, chemotherapy, radiation therapy, and hormonal therapy can damage vascular and neural structures, disrupt hormone levels, and contribute to fatigue and pain [6], as illustrated in Figure 1. Psychological factors, including anxiety, depression, body image concerns, and fear of recurrence, further worsen sexual function [6]. Sexual dysfunction affects up to 80% of men with prostate cancer [7], approximately 40–80% of women with gynecological cancers [8,9,10], and 55–73% of patients with hematologic cancers [11]. These difficulties often involve desire, arousal, or orgasm and may persist throughout survivorship [1], significantly impacting QoL, psychological well-being, and intimate relationships [12,13,14].

Despite the high prevalence of these sexual issues, many patients with cancer report receiving little to no information or guidance on how to manage them [4,14,15,16,17] due to time constraints in oncology settings, limited provider training, and discomfort in addressing sensitive topics [18]. As a result, many survivors experience unmet needs that negatively affect their overall well-being [18].

Artificial intelligence (AI) and machine learning (ML) technologies are increasingly being explored to address sexual health problems and dysfunction management [19]. Digital tools like AI and ML can analyze large, complex, and multidimensional clinical data, including patient-reported outcomes, clinical records, psychological assessments, and imaging data, to identify patterns and predict risks of SD [19]. AI-driven chatbots and virtual counselors are also being developed to provide personalized sexual health education and support in a confidential, accessible manner [20]. 

The development of these AI tools has laid the groundwork for a growing number of studies applying AI to predict treatment-related sexual health outcomes in oncology [19]. For instance, artificial neural networks (ANNs) have been used to assess the likelihood of postoperative SD in men following radical prostatectomy, offering enhanced predictive performance compared to conventional methods (Saikali et al.) [21]. In radiation oncology, deep learning (DL)-based autosegmentation models have been employed to identify and spare critical structures, such as the internal pudendal artery, an essential vessel for erectile function, thereby reducing the risk of treatment-induced sexual side effects (Balagopal et al.) [22]. While AI advancements reflect meaningful progress in addressing male sexual health following prostate cancer, the application of AI in female populations, such as those with gynecological cancers, remains significantly less developed. Disparity between the genders highlights the need for broader, gender-inclusive research to ensure that emerging technologies benefit all individuals affected by cancer-related SD.

Artificial intelligence tools offer a range of promising applications to further address sexual health in patients with cancer across the cancer care continuum, as summarized in Figure 1. The use of AI and ML for prediction, assessment, and management of SD is gaining prominence in oncological research, offering valuable tools to address sensitive and under-discussed issues. They facilitate more accurate assessments of sexual health concerns, aid in predicting the risk of future SD during survivorship, and ultimately contribute to enhancing QoL (Hanai et al., Agochukwu-Mmonu et al., Balagopal et al.) [22,23,24]. However, the integration of AI-ML in this field remains in its early stages, and further research is needed to evaluate its clinical utility, accuracy, and real-world impact. Detailed analysis and evaluation of AI models for sexual health in oncology remains an unmet need. We conducted this systematic review to fill this gap in science and oncology.

Our scientific questions in this review were:

- What are the key applications of AI-ML models in managing sexual health in oncology?
- Which AI-ML algorithms are commonly used to address sexual health and SD throughout every stage of cancer care?
- Which AI-ML models have demonstrated the highest performance in predicting sexual health outcomes?
- What is the overall quality of studies applying AI and ML techniques to sexual health in cancer care, including adherence to TRIPOD+AI guidelines for transparent and accurate model performance reporting and the percentage of risk of bias?
- To what extent are AI and ML effective in improving sexual health and predicting SD among cancer patients?

The main objective of this review was to address current gaps in knowledge by evaluating existing literature on the application of AI-ML in predicting SD and improving sexual health outcomes and QoL in individuals affected by cancer.

## 2. Materials and Methods

### 2.1. Protocol Registration

The systematic review was registered in the international prospective register database of systematic reviews (PROSPERO) on 27 February 2025 (ID number: CRD420250655313) in the context of human health care.

### 2.2. Search Strategy and Screening Process

A comprehensive systematic search of PubMed, Ovid EMBASE, and Web of Science databases was conducted for publications in English up to 18 February 2025. The concepts searched included: “artificial intelligence,” “machine learning,” “deep learning,” “neural networks,” “sexual dysfunction,” “sexual impairment,” “erectile dysfunction,” and “sexual health.” The terms were combined using AND/OR Boolean operators. Only original human studies were included; animal studies, reviews, and conference abstracts were excluded. The search strategy using Boolean operators is described in Supplementary File S1.

The screening process was conducted with two independent reviewers (VS and BG). We first screened titles and abstracts, and then screened the full text.

- Inclusion criteria: Studies eligible to be included in this systematic review had to meet all of these criteria: (1) studies applied AI or ML models in sexual health or sexual dysfunction prediction or management (i.e., either directly or indirectly related to sexual health), (2) studies in the cancer population or in the cancer care continuum (e.g., cancer screening, detection, diagnosis, treatment, or survivorship), (3) full original articles in English, (4) studies involved human subjects, and (5) models were tested for performance.
- Exclusion criteria: Articles were excluded if they met any of the following criteria: (1) the study was beyond the study aim/scope, (2) no AI or ML model was applied, (3) not an original study (i.e., review article, letter, conference abstract, editorial, or response), (4) no sexual health, sexual disease, or dysfunction (biological sex, gender, or sexual minority topics were excluded), (5) not in cancer care or oncology fields, (6) duplicate publication or correction of an original article, and (7) descriptive study that did not apply or test a model.

The primary outcome of this review was to identify the applications and performance of AI and ML models in predicting, detecting, or managing sexual dysfunction and sexual health outcomes in cancer populations across the care continuum.

### 2.3. Data Synthesis, Collection, and Analysis

The systematic review followed the Preferred Reporting Items for Systematic Reviews and Meta-Analysis (PRISMA) guidelines (checklist in Supplementary File S2). 

#### 2.3.1. Data Extraction

Data from eligible studies were extracted into standardized Excel sheets. Extracted variables included study demographics (author, year, journal, and country), study design, cancer type, AI-ML methodology, input and outcome variables, dataset characteristics, validation type, and reported model performance metrics. Performance outcomes were standardized where possible (e.g., AUC, accuracy, sensitivity, specificity, and F1 score). Where summary measures were missing or non-comparable, narrative description was applied.

#### 2.3.2. Presentation of Results

Results were summarized in tabular format to highlight study characteristics, AI methodologies, and reported outcomes. Visualizations (e.g., bar charts, pie charts, and flow diagrams) were generated in Excel to illustrate the study distribution, TRIPOD+AI adherence, cancer types, and research strategy.

#### 2.3.3. Synthesis Methods

Due to heterogeneity in study designs, cancer populations, AI models, and outcome measures, no formal meta-analysis was conducted. A narrative synthesis approach was applied, grouping studies by cancer continuum phase (screening, diagnosis, treatment, and survivorship) and AI methodology (ML, DL, and hybrid approaches). This approach was chosen to allow meaningful comparison while acknowledging methodological diversity.

#### 2.3.4. Exploring Heterogeneity

Heterogeneity was qualitatively explored by stratifying studies based on cancer type, AI model category, and validation approach.

#### 2.3.5. Sensitivity Analysis

No analyses were conducted due to the limited number and heterogeneity of eligible studies. The small sample size of included publications and the diversity of AI models and outcomes precluded meaningful sensitivity testing.

Included articles were categorized according to the applications of AI-ML models in cancer continuum phases and other applications. Excel sheets were used in collecting data, data analysis, and figure creation.

### 2.4. Data Quality and Risk of Bias

Screening of the identified articles was performed blindly by two reviewers. Full texts of the included articles were assessed thoroughly by three reviewers (BG, VS, and PL). The materials and methods of the studies and the results sections were assessed. If the study was descriptive and no results were stated, the article was excluded. Corrections, non-full articles, or full articles that could not be accessed were excluded.

To evaluate the overall quality of the included articles, a rigorous assessment was conducted, focusing on appraising both the risk of bias and adherence to reporting guidelines for each individual article. The risk of bias was assessed using the Prediction model Risk of Bias Assessment Tool (PROBAST), which examines four domains (participants, predictors, outcomes, and analysis) through 20 methodological questions to determine the overall risk of bias (checklist in Supplementary File S3) [25,26,27,28,29]. Adherence to the AI-Transparent Reporting of a Multivariable Prediction Model for Individual Prognosis or Diagnosis (TRIPOD+AI) guidelines was analyzed using the TRIPOD+AI checklist [27,30], which covers 27 items specified for AI models. The checklist is in Supplementary File S4.

## 3. Results

### 3.1. Search and Screening Results

**Figure 2:**
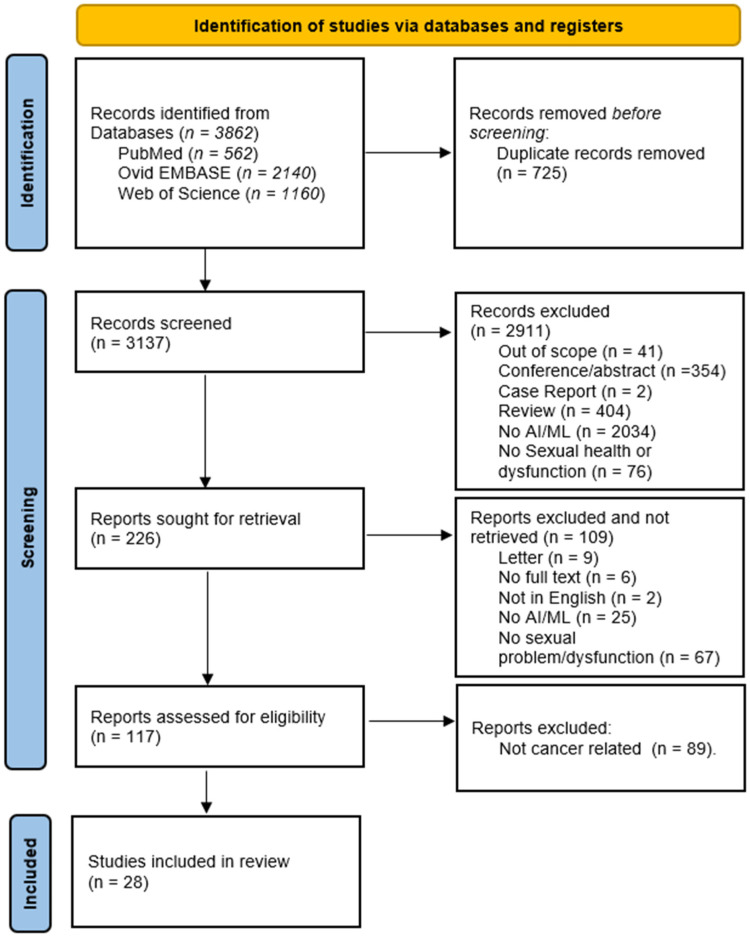
PRISMA flow diagram for systematic review of AI and ML in sexual health and dysfunction across the cancer care continuum.

Our comprehensive database search resulted in a total of 3862 studies from PubMed (*n* = 562), EMBASE (*n* = 2140), and Web of Science (*n* = 1160). After a thorough screening process following the eligibility assessment criteria, a total of 28 articles that met all the inclusion criteria and none of the exclusion criteria were included. The full search process is illustrated in the PRISMA flow chart (Figure 2).

### 3.2. Publication Trends, Design, and Populations

**Figure 3:**
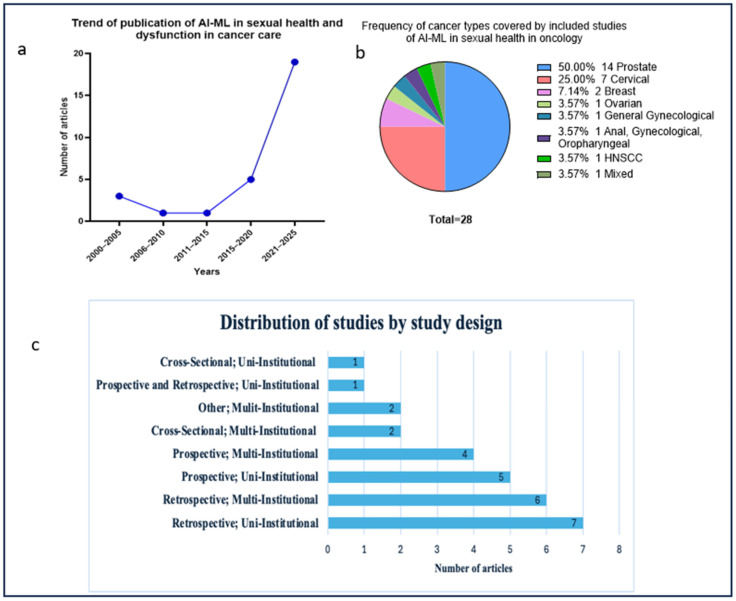
Publication trends, cancer types and designs of all included studies applying AI-ML in sexual health across cancer care. (**a**). Publication trends for studies applying AI-ML models in sexual health research across the cancer care continuum from 2000 to 2025. (**b**) Pie chart of the frequency of cancer types in included studies. (**c**) Types of studies and the number of articles per type.

All included articles were published between 2002 and 2025 (Figure 3a), with a clear upward trend in publication frequency over time. The number of studies increased from 2002–2009 (2/28) to 2010–2019 (6/28), followed by a sharp rise from 2020 to 2025 (20/28). Half of the included studies (14/28, 50%) focused on prostate cancer, making it the most frequently examined cancer type (Figure 3b). Cervical cancer was the next most common, reported in (7/28, 25%) of studies, followed by breast cancer (2/28, 7.14%). Other cancer types, including ovarian, general gynecological, head and neck squamous cell carcinoma (HNSCC), and multi-site studies involving combinations such as anal, gynecological, and oropharyngeal cancers each accounted for 3.57% (1/28). No leukemia studies were found. 

The majority of the included articles (7/28) employed a retrospective, uni-institutional design (Figure 3c). The least common designs were a mixed prospective and retrospective uni-institutional approach (*n* = 1) and a cross-sectional uni-institutional approach (*n* = 1). The median sample size across studies was 858 participants (range: 50–20,164; IQR: 1929) 95% CI 740.9–3840.5. In 15/28 (51.7%) studies, the cohort size ranged from 100 to 1000 participants, while 11/28 (37.9%) studies enrolled more than 1000 participants, and 3/28 enrolled fewer than 100 participants.

### 3.3. Artificial Intelligence and Machine Learning Models Used in Sexual Health Across Cancer Care Continuum

The demographics and characteristics of the final included studies and the AI or ML models used or developed in sexual health-related fields either directly or indirectly across the different phases or the cancer care continuum are illustrated in Table 1.

#### 3.3.1. Early Detection, Prevention, and Diagnosis Phases

Artificial intelligence tools are proposed to aid in the early identification of patients at risk for SD, including predictive modeling based on sociodemographic, behavioral, and clinical data, particularly in populations undergoing screening for reproductive/sexual organ-related cancers, such as prostate, cervical, or ovarian cancers [31,32,33,34,35,36]. Chao et al., 2022 [44] reported that a supervised ML model (gradient boosting decision tree) using clinical and demographic inputs predicted endometriosis-associated ovarian cancer (EAOC) risk, with an AUC of 0.94 (95% CI, 0.914–0.969), sensitivity of 86.8%, and specificity of 86.7%, which performed significantly better than the logistic regression (LR) model (AUC 0.89, 95% CI, 0.821–0.960). Additionally, Sun et al., 2022 [46] revealed that stacking integrated models with multiple ML algorithms (TreeBag, XGBoost, and MonMLP) improved the prediction accuracy of women at risk for cervical cancers, with an AUC of 0.877, sensitivity of 81.8%, and specificity of 81.9%. Similarly, Hariprasad et al., 2023 [48] revealed that the gradient boosting model performed better in associating risk factors with cervical cancer prediction than other ML models, with an accuracy of 98.9%. Chauhan et al., 2024 [53] also tested several ML models to predict cervical cancer risk and proved that the XGBoost model outperformed the other models, with an AUC of 0.91. Devi et al., 2024 [54] found that ensemble and DL models were effective in predicting barriers to nonattendance in cervical screening. D’Souza et al., 2010 [33] applied ML models using patient demographic and biomarker variables to predict tumor HPV16 status in head and neck cancers and revealed that the addition of HPV biomarkers improved predictions. Gentile et al., 2022 [45] applied DL (neural networks) to identify high-grade prostate cancers using the prostate health index, MRI imaging scores, and pathology results. Their model achieved a sensitivity of 80% and a specificity of 68% in diagnosing high-grade prostate cancer.

#### 3.3.2. Automatic Data Extraction

Two studies implemented natural language processing (NLP) to help extract large volumes of data from electronic health records (EHRs) and social platforms to identify unspoken or undocumented sexual health concerns (Chao et al., 2022 [44], Hernandez-Boussard et al., 2017 [36]). Hernandez-Boussard et al., 2017 [36] demonstrated that NLP algorithms were able to identify patient-centered outcomes related to ED and urinary incontinence in patients with prostate cancer pre-treatment with 85% and 87% accuracy, respectively, outperforming traditional keyword searches. Additionally, Best et al., 2018 [37] applied a digital health literacy framework to explore how patients with HPV-associated cancers access, understand, and utilize information about HPV. The study revealed that although healthcare providers were the primary source of HPV-related information, many patients exhibited limited understanding of HPV and its connection to their cancer diagnosis.

#### 3.3.3. During the Treatment Stage

Artificial intelligence and ML are valuable for real-time monitoring of sexual symptoms and supporting treatment decision making. Four studies explored AI algorithms during the cancer treatment stage. Bagshaw et al., 2021 [41] investigated a decision-making template as a web-based tool to involve prostate cancer patients in their treatment choices. The decision-making tool enabled real-time comparison of treatment modalities, predicted outcomes, and potential treatment-induced toxicities, including impacts on sexual function, thereby facilitating informed and personalized decision making. Charoenkwan et al., 2021 [42] examined multiple ML models (RF (iPMI), DT, LR, Knn, MLP, NB, SVM, and XGBoost) to predict parametrial invasion (PMI) confirmed during radical hysterectomy in patients with low-grade cervical cancer. Among these, the RF model (iPMI) achieved the highest performance, with an AUC of 0.91 (95% CI, 0.852–0.958) and an accuracy of 86%. Predicting PMI is crucial, as radical hysterectomy is an aggressive treatment associated with significant intraoperative complications, such as damage to adjacent organs and blood vessels, which can result in long-term SD.

In radiation oncology, AI and ML technologies are increasingly being leveraged to anticipate treatment-related toxicities and enhance strategies that preserve sexual function. Chan et al., 2022 [43] used an LR model to predict the severity of acute genitourinary toxicity (sexual) during RT in gynecological cancers. Additionally, Deng et al., 2023 [47] revealed that an RF-based nomogram model was effective in predicting the presence of residual tissues after LEEP surgery in cervical cancers, with an AUC of 0.98. Beyond prediction, AI could be used to optimize radiation dose planning. Balagopal et al., 2024 [22] demonstrated that DL models, particularly neural networks (NNs), can improve treatment planning by better sparing critical anatomical structures, such as sexual organs, nerves, and arteries, involved in sexual function when compared to conventional planning methods.

#### 3.3.4. Post-Treatment and Survivorship Phase

Artificial intelligence-driven longitudinal monitoring can track sexual health over time following cancer treatment. Eleven studies focused on the use of AI-ML models to predict sexual function and satisfaction in men, as well as their impact on QoL post-treatment and during the survivorship phase. Among these, eight studies applied multiple AI-ML models to predict SD or ED after radical prostatectomy (RP) in patients with prostate cancer. Bacon et al. [31], Hoffman et al. [32], Barocas et al. [35], Albers et al. [40], Hasannejadasl et al. [49], and Sibert et al. [51] used regression models (LR [31,32], longitudinal regression [35], and mixed effect model [40], LR coupled with recursive feature elimination (RFE) [49], and Lasso regression [51]), while Agochukwu-Mmonu et al., 2022 [24] demonstrated that a dynamic GBM was highly effective in predicting sexual function, achieving an AUC of 0.91. Moreover, Saikali et al., 2025 [21] revealed that an ANN showed potential in predicting erectile function 12 months following nerve-sparing robotic RP (RARP), with an AUC of 0.74. 

Van Egdom et al., 2020 [39] and Xu et al., 2023 [52] investigated several ML models for predicting patient-reported outcomes (PROs), including sexual function, post-surgery. While Van Egdom et al. found no significant association, Xu et al. revealed that ML algorithms were able to predict sexual well-being following post-mastectomy breast construction (PMBR), with an average AUC of 0.76 (95% CI, 0.70–0.83). 

Kumar et al., 2014 [34] demonstrated that an SVM-based predictive model (PrediQt-Cx) was effective in predicting QoL, including sexual function, post-treatment in cervical cancer patients. The model achieved a mean AUC of 0.90, outperforming other ML algorithms evaluated in the study.

Finally, Hanai et al., 2024 [23] applied generative AI (GPT) to deliver personalized health information on sexual health for cancer patients using epidemiological survey data on sexual difficulties among cancer survivors.

#### 3.3.5. Diagnostic Imaging Analysis

Three studies used AI-ML tools for diagnostic imaging analysis of patients with cancers of reproductive organs to preserve sexual function and optimize treatment. Hussain et al., 2021 [38] applied Bayesian networks to identify the associations between morphological features of MRI images of prostate cancers. Additionally, Lei et al., 2023 [50] revealed that a DL-based topological modulated network achieved strong performance in autosegmentation of left and right neurovascular bundles on MRI images of prostate cancer compared to expert-drawn contours, with a DSC of 81%. Similarly, Balagopal et al., 2024 [22] proved that a DL-based model (SNet-MA) achieved high performance in autosegmentation of the internal pudendal artery (IPA) on CT and MRI images of the prostate, with a DSC = 62%.

#### 3.3.6. Assessment Tools for Sexual Health in Cancer Care

The sexual assessment tools in the included studies were very diverse. The most commonly used patient-reported outcome measures (PROMs) were the EPIC-26 questionnaire (*n* = 6, 21.4%). Other PRO questionnaires used were the EORTC QLQ-30 (*n* = 3/28, 10.7%), Short Form-36 Health Status Survey (SF-36), International Index of Erectile Function (IIEF), BREAST-Q, and the Sexual Health Inventory for Men (SHIM). More than half of the studies did not report using any sexual health assessment tools (*n* = 15/28, 53.6%).

### 3.4. Types, Frequency, and Performance of Artificial Intelligence and Machine Learning Models Used in Sexual Health in Oncology

**Figure 4:**
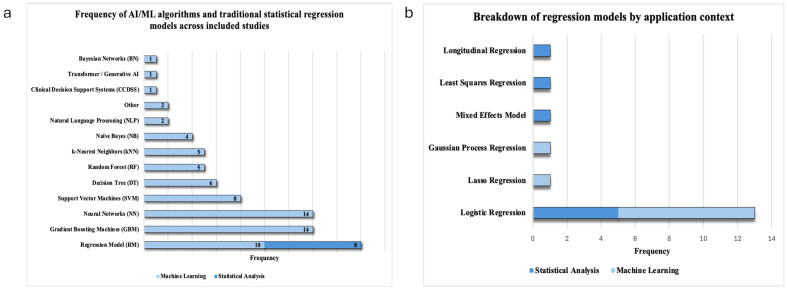
Types and frequency of AI-ML models used in sexual health across the cancer care. (**a**) Frequency of types of AI and ML models used in included studies of AI-ML in sexual health and dysfunction across the cancer care continuum. (**b**) Frequency of different types of regression models, including regression models used for traditional statistical analysis (light blue) and machine learning-based prediction (dark blue).

The most frequently used algorithm across studies was regression (*n* = 18), followed by gradient boosting machines (GBM) (*n* = 14), and neural networks (NN) (*n* = 14) (Figure 4a). Regression models were used either solely for traditional statistical analysis (*n* = 8) or for ML-based predictive analysis (*n* = 10) (Figure 4a). Other methods included support vector machine (SVM) (*n* = 8), decision tree (DT) (*n* = 6), random forest (RF) (*n* = 5), k-nearest neighbors (kNN) (*n* = 5), deep learning (DL) (*n* = 4), and naïve Bayes (NB) (*n* = 4). Less frequently used methods included AdaBoost (*n* = 3), NLP (*n* = 2), Bayesian network (BN) (*n* = 1), clinical decision support system (CDSS) (*n* = 1), and transformer-based generative AI models (*n* = 1). The “other” category (*n* = 2) included stabilized discriminant technique analysis and a digital framework.

A detailed breakdown of regression models by application context is shown in Figure 4b. Logistic regression was most common, used in both ML (*n* = 8) and traditional statistical modeling (*n* = 5). Other ML-based regressions included Lasso and Gaussian process regression with radial basis function kernels. Traditional-only models included mixed effects, least squares, and longitudinal regressions.

The performance of the AI-ML models in the studies included in this review varied based on the target outcome and application. For classification models, studies used discrimination metrics such as area under the receiver operating curve (AUC), sensitivity or recall, specificity or precision, accuracy, or F1 scores. Other studies used root mean square error (RMSE) (Hernandez-Boussard et al. [36], Agochukwu-Mmonu et al. [24], Hariprasad et al. [48], and Sibert et al. [51]), mean absolute error (MAE) (Agochukwu-Mmonu et al. [24]) or mean square error (MSE) (Kumar et al. [34]), especially in models predicting a numerical output/outcome such as PRO scores. Few articles tested the calibration of the models (*n* = 6, 21.4%) using either a calibration curve, Q–Q blots, comparing the observed mean to the mean expected scores, Hosmer–Lemeshow goodness-of-fit test, or the consistency index (C-index) (Sibert et al. [51], Chao et al, Saikali et al. [21], Deng et al. [47], Hasannejadasl et al. [49], and Agochukukwu-Mmonu et al. [24]). Studies using AI (e.g., DL- and NN-based models) for imaging analysis, such as autosegmentation, used other performance metrics, such as the Dice similarity coefficient (DSC), to test the efficacy of the models compared to the professional gold standard (clinicians used manual segmentation) (Lei et al. [50] and Balagopal et al. [22]). Studies using regression models for statistical analysis and association analysis used *p*-values, coefficient (R2), Pearson correlation, and other metrics [31,32,40,43,44,49]. Detailed performance metrics and results are summarized in Supplementary Table S1.

Performance analysis showed that ensemble models achieved better performance. Tree-based random forest had the strongest performance, with a median of AUC 0.98, (range 0.91–0.99), median sensitivity of 0.98 (range 0.60–0.98), median specificity of 0.99 (range 0.95–0.99), specificity of 0.99 (range 0.95–0.99), and F1 score of 0.99 (range 0.89–0.99). The boosting models (e.g., GBM, XGBoost, and AdaBoost) also achieved well, with a median AUC of 0.94 (range 0.77–0.99), sensitivity of 0.96 (range 0.87–0.99), specificity of 0.97 (range 0.94–0.99), and F1 score of 0.965 (range 0.94–0.99). The KNN model achieved very strong performance, with a median AUC of 0.96 (0.73–0.98). Regression models showed a lower AUC of 0.83 (0.60–0.97), sensitivity of 0.87 (0.68–0.70), and specificity of 0.78 (0.66–0.95). SVMs had the lowest performance, with an AUC of 0.77 (0.69–0.90) and sensitivity of 0.65 (0.60–0.70). See Supplementary Table S2.

### 3.5. Quality of Studies Including Adherence to Transparent Reporting of a Multivariable Prediction Model for Individual Prognosis or Diagnosis Guidelines and Risk-of-Bias Assessment

**Figure 5:**
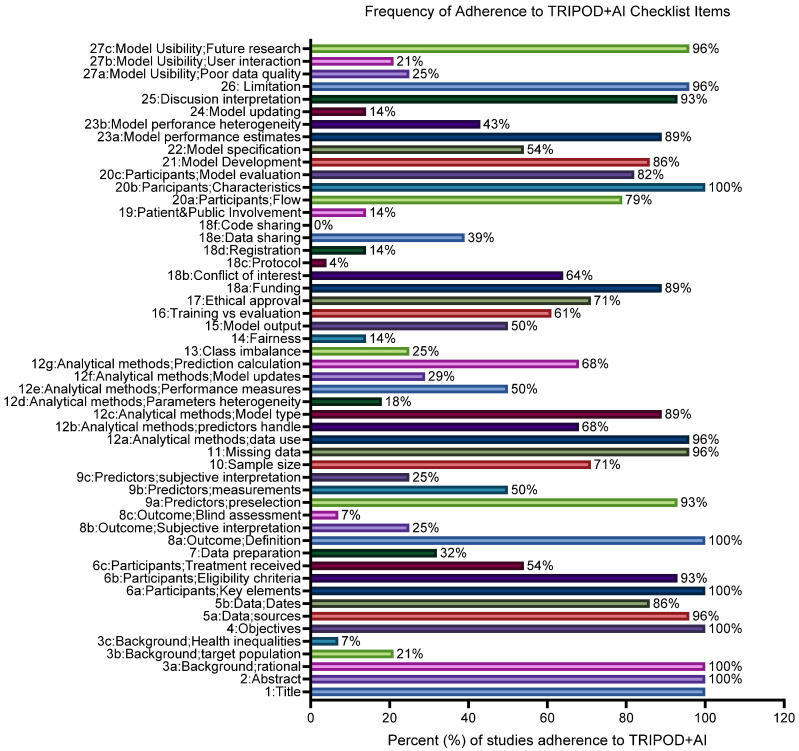
Frequency of adherence to TRIPOD+AI guidelines checklist domains across the included 28 studies in this review articles applying AI and ML in sexual health across cancer care.

The assessment of TRIPOD adherence across the included studies revealed high variability in reporting quality, as illustrated in Figure 5. The overall average adherence to the TRIPOD+AI guidelines checklist was 60% (range: 48%–73%). Several items demonstrated excellent adherence, particularly those related to study title, abstract, introduction, background rational, objectives, participant characteristics, outcome definition, model specification, and limitations, each achieving 100% (28/28) compliance. Moderate adherence was observed for treatment received (15/28, 54%), predictor measurements (14/28, 50%), model outputs (14/28, 50 differences between the training and testing datasets), and external validation (17/28, 61%). By contrast, lower reporting was observed for calibration methods such as model updating (4/28, 14%), class imbalance (7/28, 25%), dealing with parameters and performance heterogeneity (5/28, 18%), and generalizability or external validation (4/28, 14%).

Model usability, especially related to handling poor data quality and user interaction, were poorly reported (average 23%). Critical items such as data preparation, subjective interpretation of outcomes or predictors, and blind assessment were poorly reported.

Ethical approval (20/28, 71%) and conflicts of interest (18/28, 64%) were often reported, but protocol registration (4/28, 14%), data sharing (11/28, 39%), and code sharing (0/28, 0%) were rarely addressed. Decision curve analysis and clinical utility were almost absent (0–4/28, 0–14%), and blinding of outcome assessment was reported in only 2/28 (7%) studies.

Of the 28 included studies, the overall risk of bias was high in all 28 studies (100%). In the participant, predictor, and outcome domains, all 28 studies (100%) were rated as low risk, indicating clear eligibility criteria, well-defined predictors available at the time of model use, and appropriately defined and measured outcomes. However, every study (28/28; 100%) demonstrated a high risk of bias in the analysis domain.

## 4. Discussion

Sexual health remains a critical but underrecognized aspect of oncology care, with significant implications for QoL. Our systematic review demonstrated that while digital technologies such as AI and ML tools are being applied across different stages of the cancer care continuum, high-quality studies are still limited and characterized by important methodological and clinical gaps. The included studies highlighted the applications of AI and ML during screening and early detection of cancers of reproductive organs affecting sexual dysfunction during cancer treatment and post-cancer survivorship. The various applications of AI in sexual health showed the potential of these technologies in sexual health care in oncology; however, rigorous studies are still needed for more accurate and reliable AI models. In recent years, AI digital technologies, particularly NLP and imaging-based tools, have been increasingly employed to automate complex and time-consuming tasks in cancer care. Several studies in this review utilized NLP algorithms to extract relevant PROs and clinical data from unstructured sources such as EHRs, clinical notes, and narrative surveys. Data extraction automation facilitates large-scale analysis of sexual health symptoms that are often under-documented or inconsistently reported in structured data fields. Similarly, AI-driven imaging analysis and auto-segmentation tools have been integrated into radiation oncology workflows to delineate organs at risk with high precision in several cancer types. Choi et al., 2023 [55] proved the utility of DL-based auto-contours of organs at risk in breast cancer patients. In our review, studies used DL- and NN-based tools for imaging analysis and autosegmentation of structures at risk in prostate cancers, including structures related to sexual function (e.g., neurovascular bundles and interparental arteries). Deep learning and neural network tools improve consistency, reduce interobserver variability, and enable personalized radiation planning that can mitigate treatment-related SD in prostate cancers. However, no studies in our review addressed autosegmentation of structures at risk in gynecological or anal cancers, which requires further research.

Most studies applying AI included male patients with prostate cancer, and this concentration reflects both the prevalence of this cancer and the availability of data, but it also highlights a research imbalance. Few studies addressed female gynecological cancers beyond the cervix, and none examined populations affected by sexual health disparities, such as survivors of anal, ovarian, or hematologic malignancies. The discrepancy in cancer types raises concerns about the inclusivity and generalizability of AI-driven solutions, emphasizing the need for more research in other cancer types, particularly those affecting women (gynecological cancers) and underserved populations.

Several recent studies investigated the efficacy of ML models for predicting SD or ED. Chen et al., 2024 [56] revealed that a gradient boosting-based model (XGBoost) was effective in ED prediction in males using multidimensional features extracted from the National Health and Nutrition Examination Survey (NHANES), with an AUC of 0.89. Chen et al.’s results align with our results, which revealed that gradient boosting-based models are the most common ML models used and ensemble methods consistently delivered high performance, indicating strong discrimination capabilities and better dealing with multidimensional data with nonlinearity. Our results revealed that the strongest predictive performance was consistently achieved by ensemble methods such as random forest and boosting models. These models are well-suited to capturing nonlinear relationships and integrating multidimensional data (e.g., clinical, sociodemographic, and imaging). Moreover, ML often outperformed traditional statistical approaches, especially in dealing with multidimensional variables for sexual function prediction.

Our review revealed significant heterogeneity and limited use of standardized tools to assess sexual function in cancer patients. Most studies lacked validated, cancer-specific instruments, making comparisons across studies difficult and limiting the clinical applicability of AI-ML models. More than half of the included studies relied on general tools, which were developed outside oncology contexts and fail to capture cancer-specific challenges, such as treatment-induced dyspareunia, vaginal dryness, or body image distress. Even models reporting excellent discrimination were built on outcomes measured with blunt or incomplete tools, raising concerns about construction validity. Without standardized, validated measures, it is difficult to benchmark performance, replicate findings, or translate AI-driven insights into meaningful interventions for patients. The same gaps were also noted in prior reviews (Eeltink et al., 2022 [17]; Rodrigues-Machado et al., 2025 [57]), highlighting the need for a validated, cancer-specific PRO measure that comprehensively assesses sexual health across cancer care to improve both research consistency and clinical guidance.

Suboptimal adherence to TRIPOD+AI guidelines with low compliance in key domains revealed in the results of our study, in addition to lack of model calibration assessment, raise great concerns. Stricter adherence to TRIPOD+AI guidelines is needed, especially in studies leveraging AI and ML in oncology, to ensure transparency, replicability, and trust in predictive model development and evaluation [27,28,29,30]. Our findings also highlighted important ethical considerations in applying AI to sexual health in cancer, where ethical approval was moderately addressed. Critically, all studies were assessed as having a high risk of bias, revealing analytical limitations across these studies such as overfitting, lack of calibration testing, and absence of external validation. 

Deficiencies in reporting and the quality of AI studies have direct implications for the generalizability and clinical applications of AI-ML models. Without calibration, models may systematically over- or under-estimate risks, making them unreliable in diverse clinical settings. The lack of external validation further undermines confidence in the generalizability of the model and the reproducibility of findings across populations, institutions, or cancer types. Poor handling of missing data and inadequate transparency in reporting limit replicability, making it difficult for clinicians or decision makers to judge the readiness of these tools for practice. In short, even models with high reported performance cannot be confidently translated into real-world oncology care if these methodological weaknesses are not addressed [17,52]. Moreover, the sensitive nature of sexual health data underscores the need for rigorous safeguards around data privacy and informed consent. Future work must balance the opportunities of using novel AI tools with adherence to reporting guidelines and ethical caution to ensure that implementation is equitable, transparent, and respectful of patients’ rights and sensitivities.

To enable clinical adoption, healthcare providers need structured guidance on integrating AI-ML into sexual health care within oncology. Validated AI tools can support early risk stratification of sexual dysfunction, timely referrals, and EHR integration to prompt routine discussions. Artificial intelligence-based decision support can identify high-risk patients, such as in radiation oncology, by using autosegmentation to preserve sexual organs and supportive structures. Natural language processing-derived PROs allow real-time monitoring for early intervention. Artificial intelligence tools should support clinical judgment, promoting personalized and equitable care. It is crucial that these tools are rigorously developed and validated through transparent, high-quality studies to ensure their reliability, usability, and acceptance by clinicians and healthcare providers. Multidisciplinary guidelines are needed to standardize use of these digital tools across cancers, settings, and populations, ensuring benefits while minimizing harm.

### 4.1. Future Studies 

Future studies must prioritize methodological rigor in applying AI and digital technologies in sexual health care in oncology, ensuring external validation, calibration, and transparent reporting for future clinical applications. Additionally, using standardized outcomes and validated, cancer-specific sexual health measures is very critical for the accuracy and reliability of the models. Moreover, equity and inclusivity through expanding research to cancers affecting women and underserved populations is needed in future studies to increase AI generalizability.

### 4.2. Limitations

The limitations of this review include, first, despite employing a comprehensive search strategy across multiple databases, the number of studies specifically applying AI or ML to sexual health in oncology was limited, highlighting the nascent and underexplored nature of this field. Consequently, studies involving AI applications with potential indirect effects on sexual health (e.g., in reproductive organ cancers) were also included. Second, heterogeneity in cancer types, AI methodologies, models evaluation metrics, patient populations, and sexual health assessment tools hindered a formal meta-analysis and limited the comparability of study outcomes. Third, many included studies lacked external validation and had methodological shortcomings, such as small sample sizes, unclear handling of missing data, and insufficient details regarding model calibration and blinding procedures, which may compromise reproducibility and generalizability. Fourth, over half of the studies did not use validated tools for measuring sexual function or QoL, potentially introducing outcome measurement bias. Finally, publication bias cannot be excluded, as studies with negative or less promising findings may be underrepresented in the published literature.

## 5. Conclusions

Artificial intelligence and machine learning tools hold potential for advancing sexual health care in oncology across all phases of the cancer care continuum, with demonstrated applications in risk prediction, monitoring, and treatment planning. However, the current evidence is constrained by methodological limitations, poor adherence to reporting standards, lack of external validation, and inconsistent or non-validated sexual health measures. Rigorous, high-quality studies are required, especially in AI applications in cancer care for clinical applications. 

## Figures and Tables

**Table 1:** Demographics and characteristics of included studies.

Author et al., Year	Journal	Type of Cancer	Number of Population/ Participant	AI-ML Type	Input Variables	Output Outcome	Conclusion	Sexual Health Assessment Tools	Application in Cancer Care
Bacon et al., 2002 [31]	*Cancer An international Interdisciplinary Journal of the American Cancer Society*	Prostate	783	Least square regression models. LR models	Sexual function, urinary function, bowel function, covariates: age, marital status, waist circumference, physical activity, smoking status, alcohol intake, comorbid conditions	Quality of life: general health-related and cancer-specific. Symptom bother: UCLA bother scales and SF-36 scales: impact of sexual, urinary, and bowel symptoms on overall and cancer-specific quality of life of prostate cancer patients.	Prostate cancer patients reported significantly greater bother and effect on QOL from sexual, urinary, and bowel symptoms. Sexual symptoms were strongly associated with worse QoL.	SF-36 CARES-SF UCLA Prostate Cancer Index (to assess sexual symptoms related to prostate cancer therapy)	Cancer survivorship
Hoffman et al., 2003 [32]	*Cancer*	Prostate	2365	LR	Age, race/ethnicity, treatment type (active vs. conservative), perception of being cancer-free, urinary and bowel functions, erectile function, general health status, and social support measures	Patient satisfaction with treatment decision two years after diagnosis with prostate cancer.	Most men were satisfied with their treatment selection for clinically localized prostate carcinoma. Men tended to minimize loss of urinary, bowel, or sexual functions by not perceiving dysfunction as a problem.	None reported	Post-treatment survivorship
D’Souza et al., 2010 [33]	*Oral Oncol*	Head and Neck (HNSCC)	255	DT SVM	Demographics: age, gender, race/ethnicity. Behavioral: tobacco use, alcohol use, sexual behavior (# of oral sex partners), income, education. Biomarkers: HPV16 L1 E6/E7, HPV16 DNA	Prediction of tumor HPV16 status (positive vs. negative) in HNSCC as determined by in situ hybridization	Models used only demographics (tobacco use, age, gender, race) with moderate predictive values: HNSCC: PPV = 75%, NPV = 68%; Oropharyngeal cancers only: PPV = 55%, NPV = 66%. Addition of HPV biomarkers improved predictions	None reported	Cancer prediction
Kumar, et al., 2014 [34]	*PLoS ONE*	Cervical	198	SVM with both linear and RBF kernels, multilayer perceptron (ANN), and regularized LR"	13 variables: age, marital status, vaginal bleeding, vaginal discharge, dyspareunia, abdominal pain, weight loss, parity, bowel or bladder control difficulty, cervical cancer stage, treatment modality, lymphoedema, and peripheral neuropathy.	Three post-treatment HRQoL outcomes (including sexual function), at 6 months post-treatment in cervical cancers.	The prediction model (PrediQt-Cx), based on support vector machine (SVM) for predicting post-treatment HRQoL in cervical cancer patients was developed and internally cross validated. The performance of SVM (linear) exceeded other models in most domains (mean AUC of 0.90). Patients experienced substantial decrease in sexual activity post-treatment.	EORTC QLQ C-30 and CX-24, (HRQoL) questionnaires,	Post-treatment survivorship
Barocas et al., 2017 [35]	*JAMA*	Prostate	2550	Longitudinal regression model	Baseline domain scores (EPIC-26), age, race/ethnicity, comorbidity index, prostate cancer risk stratum (D’Amico classification), physical function (SF-36), social support, depression score, medical decision-making style, treatment type (radical prostatectomy, EBRT, active surveillance)	Patient-reported functional outcomes (EPIC-26 domains: sexual, urinary incontinence, urinary irritative, bowel, hormonal); health-related QoL (SF-36); disease-specific and overall survival after prostate cancer treatment.	Radical prostatectomy was associated with worse sexual function and urinary incontinence at 3 years compared to EBRT and active surveillance. Radical prostatectomy was associated with improved urinary irritative symptoms. No meaningful long-term differences in bowel or hormonal function. No significant differences in disease-specific survival (≥99.7%)	EPIC-26	Post-treatment survivorship
Hernandez-Boussard, T., 2017 [36]	*AMIA Annu Symp Proc*	Prostate	7109	Natural Language Processing	Patients’ demographics, health care encounters, diagnosis/problem lists, unstructured EHR clinical notes/documents, lab & diagnosis results, ICD codes, CPT codes, medications, patient history, text features (affirmed, negated, risk discussion mentions of urinary incontinence and ED)	Extraction and classification of patient-centered outcomes (urinary incontinence and erectile dysfunction) from clinical notes of EHR.	"NLP pipeline developed and validated for detecting clinical mentions of patient-centered outcomes in prostate cancer patients, including UI and ED. This system showed performance sufficient for health management and treatment decisions."	"ICD-9/ICD-10, billing codes, medications, and vocabularies matched with existing ontologies from the NCBO and UMLS concepts"	Data extraction EHR
Best, A. L., 2018 [37]	*Journal of Health Communication*	Anal, gynecological, and oropharyngeal	50	Applied thematic framework and digital application	Sociodemographic of respondents (age, education level, household income, gender, sexual orientation, and marital status). Attitudes toward digital social prescribing, perceived benefits and challenges, technology acceptance metrics	Assessment, understanding, appraising, and applying cancer patients’ HPV information in the context of their cancer diagnosis. Perceptions of digital social prescribing platforms and identification of facilitators and barriers to implementation in mental health services	"The health literacy framework and the digital applications used to explore how patients diagnosed with HPV-associated cancers accessed, understood, appraised, and applied HPV information.	None reported	Data extraction STD diagnosis
Hussain et al., 2019 [38]	*IEEE Access*	Prostate	682	Bayesian Networks	MRI prostate cancer images, including morphological features: variables such as area, equidiameter, circulatory 1 and 2, elongatedness, entropy, maximum radius, minimum radius, and eccentricity	The associations between several key morphological features extracted from prostate cancer images.	"A Bayesian network quantified the association between different imaging morphological features of prostate cancers.	None reported	Imaging analysis
van Egdom et al., 2020 [39]	*Breast J*	Breast	764	GLM SVM ANN DL	Age, medical status, tumor characteristics, and possible (neo)adjuvant treatment indications/treatment characteristics	Prediction of patient-reported outcomes (PROs) post-surgery, including HRQoL, sexual, physical, and psychosocial function	No significant predictive relationship was found; model accuracy aligned with outcome prevalence, not effective for individual-level prediction.	EORTC QLQ-C30 EORTC QLQ-BR23 BREAST-Q	Post-treatment survivorship
Albers et al., 2021 [40]	*J Sex Med (The Journal of Sexual Medicine)*	Prostate	884	Mixed effect model (regression model) Multiple LR	Age, baseline IIEF-5 score, baseline overall satisfaction score, sexual desire score, IPSS, incontinence score, fascia preservation score, QoL	"ED due to robot-assisted radical prostatectomy (RARP). Primary: Overall satisfaction with sexual life (from IIEF-15 Q13+Q14) Secondary: Factors associated with satisfaction at 24 and 36 months"	The model proved that no increase or decrease in overall satisfaction with sexual life between 6 m and 36 m follow-up after RARP, while a higher overall satisfaction at baseline and high sexual desire were associated with satisfaction at 24 and 26 m follow-up. Erectile function score was not correlated with overall satisfaction.	IIEF-15 IIEF-5	Post-treatment survivorship
Bagshaw et al., 2021 [41]	*BMC Medical Informatics and Decision Making*	Prostate	750	Decision-making template (web-based decision aid)	NCCN risk group, pre-treatment health state (ED, urinary incontinence, nocturia, bowel incontinence), treatment options (AS, SBRT, EBRT ± HDR ± ADT), 5-year biochemical control rate, patient preference thresholds, willingness-to-be-paid values	Prostate cancer patients’ treatment decisions and engagement. [Personalized ranking of treatment alternatives based on individual risk, preferences, side effect tolerances, and value assessment]	Web-based decision aid was successfully built; visual interface allowed real-time updates based on patient preferences. Every treatment could be optimal for different individuals. No single dominant option across all users.	EPIC questionnaire	Cancer treatment
Charoenkw et al., 2021 [42]	*Diagnostics*	Cervical	1112	RF-classifier, named iPMI LR DT kNN MLP NB SVM XGBoost	The initial model, iPMI-Econ: basic clinical and pathological features before surgery: demographics, tumor characteristics, and clinical staging.	Presence or absence of parametrial invasion (PMI) was confirmed during surgery.	iPMI-Power was effective and had superior performance compared to other well-known ML classifiers in predicting PMI.	None reported	Cancer treatment
Agochukwu-Mmonu et al., 2022 [24]	*European Urology Open Science*	Prostate	2653	GBDT	Clinical and demographic features: age, race, BMI, diabetes, PSA, T stage, Gleason grade, prostate volume, baseline/post-op EPIC-26 sexual domain score, PROMIS satisfaction with sex life, use of erectile aids, nerve-sparing status, surgical volume	Sexual function, sexual activity, and satisfaction with sexual life at 3, 6, 12, and 24 months after radical prostatectomy (RP). EPIC-26 sexual domain score, EPIC-26 dichotomized score ≥73, erection quality at 12 and 24 months	A dynamic ML model (GBM) was developed and validated to predict sexual function before and after RP. GBM achieved high performance for preoperative predictions and even better performance for dynamic predictions	EPIC-26. PROMIS Interest in “Sexual Activity and Global Satisfaction with Sex Life subdomains"	Post-treatment survivorship
Chan et al., 2022 [43]	*Current Oncology (Curr Oncol)*	Gynecologic	698	LR	Patient demographics (age, cancer type, concurrent chemotherapy, radiation technique), baseline and weekly patient-reported symptom scores from a 49-item PRO questionnaire covering GI, GU, bowel, urinary, abdominal, gynecological, sexual/vaginal, and general health domains.	Acute toxicity trends, specifically GI and GU toxicity severity, during RT	PRO data can be used to track acute toxicity (including GU and sexual activity) during RT in gynecological cancers. Patients were approximately 6 times less likely to respond to questions about vaginal and sexual health after treatment	"PRO questionnaire. EPIC Bowel/Urinary 2.0, PRO-CTCAE GI, EORTC QLQ CX24, and EuroQol EQ-5D-5L"	Cancer treatment
Chao et al., 2022 [44]	*Acta Obstetricia et Gynecologica Scandinavica*	Ovarian	6809	NLP for data extraction from EMR GBDT LR	A total of 94 demographic and clinicopathologic variables for GBM. For LR model: 10 selected features (age, dysmenorrhea, GnRHa before surgery, FH of EC, hist of cystectomy, Mirena, serum CA125, max tumor diameter, leiomyoma, peritoneal endometriosis).	Endometriosis-associated ovarian cancer (EAOC) [which significantly affected sexual health/activity and induced pain]	ML-based risk model was constructed to predict endometriosis EAOC, which had high sensitivity and specificity. The ML model performed significantly better than the LR model	None reported	Cancer prediction and diagnosis
Gentile et al., 2022 [45]	*Clinical Genitourinary Cancer*	Prostate	135	NN (DL)	Prostate Health Index (PHI), PI-RADS score from multiparametric MRI (mpMRI), Gleason score from pathology	Identify high-grade prostate cancer. Binary classification: clinically significant prostate cancer (csPCa) vs. indolent PCa (based on Gleason score ≥7 or 7 with pattern 4)	Deep learning combining mpMRI and PHI may help to better estimate the risk category of PCa at initial diagnosis	None reported	Cancer diagnosis
Sun et al., 2022 [46]	*Frontiers in Oncology*	Cervical	858	Stacking integrated ML algorithm that combined multiple base models, including RF, SGB, TreeBag, XGBoost, MonMLP, SVMRadial, KNN, GaussPrRadial, RgeLogistic, SLDA, LMT	Hormonal contraceptives (years), number of pregnancies, smoking (years), number of cigarette packets smoked annually, number of sexual partners, the use of an intrauterine device (IUD) (years), number of sexually transmitted diseases (STDs), human immunodeficiency virus (HIV), and age.	Accurate identification of women at high risk of cervical cancer	A stacking integrated model that incorporated multiple algorithms was developed to improve prediction accuracy of women at high risk for cervical cancer. The stacking integrated model with TreeBag, XGBoost, and MonMLP as base classifiers and LMT as the result classifier showed the best performance.	None reported	Cancer prediction/prevention
Deng et al., 2023 [47]	*Frontiers in Medicine*	CIN3 (cervical)	436	Multivariate LR analysis for independent risk factors RF for prediction	Preoperative ECC pathology, LEEP margin status, post-LEEP follow-up HPV, post-LEEP follow-up Thin Prep Cytology Test (TCT), and gland involvement	Presence or absence of residual lesions confirmed by histopathology after total hysterectomy performed within three months post-LEEP.	The post-LEEP follow-up HPV, post-LEEP follow-up TCT, and gland involvement are independent risk factors related to residual tissue after LEEP surgery in CIN3. The constructed RF-based nomogram model effectively predicted the presence of residual tissue after LEEP surgery in CIN3	None reported	Cancer treatment
Hariprasad et al., 2024 [48]	*IEEE Access*	Cervical	858	GBM XGBoost RF SVM MLP KNN LR	Age, number of sexual partners, age at first sexual intercourse, smoking status, hormonal contraceptive use, number of pregnancies, history of STIs, IUD, and other gynecological factors	Risk of cervical cancer	The gradient boosting model was effective in associating risk factors with cervical cancer prediction, with an accuracy of 98.9%	None reported	Cancer prediction/prevention
Hasannejadasl et al., 2023 [49]	*PLoS ONE*	Prostate	964	Logistic regression algorithm coupled with recursive feature elimination (RFE).	(i) At diagnosis: PROMs data from the EPIC26 questionnaire; (ii) tumor characteristics, such as tumor staging, PSA at diagnosis, and ISUP Gleason grade group; (iii) patient characteristics: age, height, weight, smoking, comorbidities, and treatment	Erectile dysfunction (the frequency of erections) at 1 year and 2 years post-diagnosis with prostate cancer.	Two models generated using an LR algorithm coupled with RFE revealed high AUC (0.84 and 0.81 for 1 year and 2 years, respectively) for prediction of ED post-diagnosis of prostate cancer.	EPIC-26 questionnaire	Cancer survivorship
Lei et al., 2023 [50]	*Med Phys*	Prostate	60	DL-based topological modulated network	MRI images	Automatic segmentation of the left and right neurovascular bundles (NVBs) on MRI.	The topological modulated network achieved strong performance in segmenting both left and right NVBs when compared to expert-drawn contours	None reported	Imaging analysis
Sibert et al., 2023 [51]	*PLoS ONE*	Prostate	20164	Lasso regression, kNN	Baseline and follow-up EPIC-26 sexual function scores, age, baseline function, hormone therapy use, dosimetric parameters, comorbidities	Urinary incontinence and sexual function scores at one year after radical prostatectomy (RP), as assessed by EPIC-26.	Lasso regression model was developed and validated to predict functional outcomes (sexual function and incontinence) 1 y after RP. Models showed appropriate predictive properties	EPIC-26 questionnaire	Post-treatment survivorship
Xu et al., 2023 [52]	*Ann Surg Oncol*	Breast	1454	LR with elastic net XGBoost NN	BMI, preoperative BREAST-Q scores for physical well-being, sexual well-being, and psychosocial well-being; reconstruction technique, ALND, SLNB, socioeconomic status; race; education level; working status	Predict changes in physical, sexual, and psychosocial well-being two years following PMBR	ML models are capable of accurately predicting long-term PRO after postmastectomy breast reconstruction	BREAST-Q	Post-treatment survivorship
Balagopal et al., 2024 [22]	*Physics & Imaging in Radiation Oncology*	Prostate	86	DL	Imaging modality (CT and MRI), segmentation metrics (DSC, ASD, and HD95), dose-volume parameters (Dmean, V20), observer contour quality scores	Internal pudendal artery (IPA) segmentation quality for preserving sexual potency, dosimetric similarity, and improvement in physician contouring efficiency and quality	A DL model for good-quality IPA contours with DSC 62.2%, which improved uniformity of segmentation and facilitating standardized IPA segmentation in clinical trials and practice	None reported	Imaging analysis
Chauhan et al., 2024 [53]	*MethodsX*	Cervical	858	CHAMP (cervical health assessment using machine learning for prediction) included multiple ML, including XGBoost SVM NB AdaBoost DT KNN	Age, HPV results, cytology outcome, number of sexual partners, smoking, hormonal contraceptive use, IUD use, STDs, etc.	Prediction of cervical cancer risk (binary classification)	The study tested several ML models to predict cervical cancer risk. XGBoost model performed the best. It showed high accuracy and reliability compared to other models like SV and NB	None reported	Cancer prediction/prevention
Devi et al., 2024 [54]	*Public Health Nursing*	Cervical	1046	Classification Models: NB LR Ensemble Models: RF, Bagging, Soft Voting, Weighted Averaging DL Models: MLP, NN, Long Short-Term Memory LSTM	22 predictors	Prediction of nonattendance to cervical cancer screening	Employing ensemble and DL models proved most effective in predicting barriers to nonattendance in cervical screening	None reported	Cancer screening/prevention
Hanai et al., 2024 [23]	*BMJ Health & Care Informatics*	Mixed	100	Generative AI (GPT)	Epidemiological surveys data regarding sexual difficulties among cancer survivors. [The prompt ‘I am a cancer survivor. Please create a question about a problem that is hard to consult’ generated 100 questions by DocsBot that had learnt a survey on sexual problems among cancer survivors.]"	Generated questions categorized into 7 topics based on the symptom categories specified in the clinical guidelines: sexual response, body image, intimacy, sexual functioning, vasomotor symptoms, genital symptoms, and others. Distribution and content of AI-generated answers regarding sexual health in cancer survivors	"Generative AI can serve to provide health information on sensitive topics such as sexual health"	None reported	Data Extraction/Cancer survivorship
Saikali et al., 2025 [21]	*Computer Methods and Programs in Biomedicine*	Prostate	8524	Neural Network (ANN), SVM, XG Boost	Urinary continence: patient age, BMI, prostate size, and baseline urinary symptom severity as measured by IPSS. Clinical factors: prior (TURP), tumor stage, prostate volume, and the Charlson Comorbidity Index. Erectile function: age, BMI, Gleason score, diabetes, baseline SHIM scores	Prediction of urinary control and erectile function following nerve-sparing robotic radical prostatectomy RARP (12 ms post-op)	AI-based models (ANN) show potential in predicting postoperative functional outcomes (potency (sexual function) and continence) following RARP	Sexual Health Inventory of Men (SHIM)	Post-treatment survivorship

## Data Availability

All data extracted from included studies, including the data collection template, are available from the corresponding author upon reasonable request. Data of AI models performance, risk of bias, and TRIPOD+AI are included in Supplementary File S5. No analytical code or additional materials were generated or made publicly available for this review.

## Supplementary Materials

The following supporting information can be downloaded at: https://www.mdpi.com/article/10.3390/cancers17183025/s1, Table S1: Performance metrics and results of AI-ML models in included articles applied AI and ML in sexual health in cancer care continuum, Table S2: Median performance metrics scores among different models, File S1: Research strategy of databases using Boolean operators, File S2: PRISMA 2020 Checklist and location where item is reported in our systematic review, File S3: PROBAST, Prediction model study Risk Of Bias Assessment Tool, File S4: TRIPOD+AI Checklist, File S5: Data Extraction and Analysis.

## Abbreviations

QoL	Quality of life
SD	Sexual dysfunction
PRO	Patient-reported outcome
STI	Sexually transmitted infections
IUD	Intrauterine device
ALND	Axillary lymph node dissection
SLNB	Sentinel lymph node biopsy
TURP	Prior transurethral resection of the prostate
HPV	Human papilloma virus
SF-36	Medical Outcomes Study Short Form–36 Health Status Survey
CARES-SF	Cancer Rehabilitation Evaluation System–Short Form
UCLA	University of California at Los Angeles
HRQoL	Health-related quality of life
EPIC-26	Expanded Prostate Cancer Index Composite-26
SHIM	Sexual Health Inventory of Men
EORTC	European Organization for Research and Treatment of Cancer
QLQ C-30	Quality of Life questionnaire
CX-24	Cervical Cancer-24
IIEF-15	International Index of Erectile Function-15
IIEF-5	International Index of Erectile Function 5
PROMIS	Patient-Reported Measurement Information System
NCBO	National Center for Biomedical Ontology
UMLS	Unified Medical Language System
ISUP	International Society of Urological Pathology
BREAST-Q	Breast-related Quality of life Questionnaire
IPSS	International Prostate Symptom Score
LR	Logistic regression
DT	Decision tree
SVM	Support vector machine
ANN	Artificial neural network
MLP	Multilayer perceptron
NLP	Natural language processing
GLM	General linear model
RF	Random forest
kNN	k-Nearest neighbor
NB	Naive Bayes
XGB-XGBoost	Extreme gradient boosting
GBDT	Gradient boosting decision tree
GBM	Gradient boosting model
SGB	Stochastic gradient boosting
MonMLP	Monotone multilayer perceptron neural network
SVMRadial	Support vector machine with radial basis function kernel
GaussPrRadial	Gaussian process with radial basis function kernel
SLDA	Stabilized linear discriminant
AdaBoost	AdaBoost classification tree
LMT	Logistic model tree
TreeBag	Bagged classification and regression tree
RgeLogistic	Regularized logistic regression
DL	Deep learning
NN	Neural network
FCN	Topological fully convolutional network
LSTM	Long short-term memory
