# Supplementary material for: Artificial Intelligence and Machine Learning in Sexual Health and Dysfunction Across the Cancer Care Continuum: A Systematic Review
Source: Cancers (Basel). 2025 Sep 16;17(18):3025. doi: 10.3390/cancers17183025 (PMC12468962; doi:10.3390/cancers17183025)
Supplement: Supplementary file 1 [file cancers-17-03025-s001.zip › cancers-3865788-xml-suppl-done.pdf]

cancers

MDPI

Supplementary Table S1. Performance metrics and results of AI-ML models in included articles applied AI and ML in sexual health in cancer care continuum.

Author, year	Model	ROC-AUC (95% CI)	Sensitivity/Recall	Specificity/precision	Accuracy	F1-Score	Root mean squared error (RMSE)	Mean absolute error (MAE)	Other measures	Calibration
Bacon et al, 2002	NLP pipelines using Empath and LIWC; rule-based classifier	Not reported	Not reported	Not reported	Not reported	Not reported	Not reported	Not reported	p values, Beta, SE and r2	Not reported
Hoffman et al., 2003	Logistic Regression	Not Reported	Not Reported	Not Reported	Not Reported	Not Reported	Not Reported	Not Reported	"P values and probability of being satisfied. 59.2% of subjects were delighted or very pleased with their treatment selection.	Not Reported
D'Souza et al, 2010	Decision Tree (main model); SVM	Not Reported	OPC: highest: 87% HNSCC: highest: 73%	OPC: highest: 41% HNSCC: highest 97%	Not Reported	Not Reported	Not Reported	Not Reported	Positive Predictive Value (PPV) for OPC: 60%, for HNSCC: 83%. Negative Predictive Value (NPV)	Not reported

 https://doi.org/10.3390/10.3390/cancers17183025

									for OPC: 77% and for HNSCC: 85%	
Kumar et al., 2014	SVM with both linear and RBF kernels, MLP (ANN),regularized LR	"Symptom Scale SVM (Linear): 0.90 SVM (RBF): 0.80 LR: 0.72 ANN: 0.85 Global Health/QoL SVM (Linear): 0.84 SVM (RBF): 0.80 LR: 0.64 ANN: 0.73 Functional Scale SVM (Linear): 0.85 SVM (RBF): 0.90 LR: 0.60	Not Reported	Not Reported	"Symptom Scale SVM (Linear): 97.37% SVM (RBF): 94.58% LR: 94.34% ANN: 87.56% Global Health/QoL SVM (Linear): 95.26% SVM (RBF): 93.12% LR: 89.29% ANN: 74.38% Functional Scale SVM (Linear): 95.81% SVM (RBF): 97.32% LR: 93.12%	Not Reported	Not Reported	Not Reported	"Mean square Error (MSE) MSE Symptom Scale SVM (Linear): 0.02 SVM (RBF): 0.03 LR: 0.02 ANN: 0.03 MSE Global Health/QoL SVM (Linear): 0.07 SVM (RBF): 0.08 LR: 0.13 ANN: 0.08 MSE Functional Scale SVM (Linear): 0.13	Not Reported

		ANN: 0.83"			ANN: 71.28%"				SVM (RBF): 0.26<br>LR: 0.16<br>ANN: 0.13<br><br>AMI Symptom Scale<br>SVM (Linear): 0.92<br>SVM (RBF): 0.81<br>LR: 0.82<br>ANN: 0.82<br><br>AMIGlobal Health/QoL<br>SVM (Linear): 0.79<br>SVM (RBF): 0.20<br>LR: 0.59<br>ANN: 0.65<br><br>AMI Functional Scale<br>SVM (Linear): 0.77<br>SVM (RBF): 0.78	

									LR: 0.34 ANN: 0.90 "	
Barocas et al., 2017	Generalized estimating equations (GEE) for longitudinal regression with regression splines	Not reported	Not reported	Not reported	Not reported	Not reported	Not reported	Not reported	generalized estimating equations (GEE) with a independent weight matrix) for association.	Not reported
Hernandez-Boussard et al., 2017	NLP	Not Reported	Urinary Incontinence: 0.87 Erectile/Sexual Dysfunction: 0.84	Not Reported	UI: 0.87. ED: 0.86	F-measure accuracy: 87%, 85%	UI: 0.87. ED: 0.85	Not Reported	Not Reported	Not Reported
Best et al, 2018	Health literacy framework	Not reported	Not reported	Not reported	Not reported	Not reported	Not reported	Not reported	Not reported	Not reported
Hussain et al., 2019	Bayesian Networks	Not Reported	Not Reported	Not Reported	Not Reported	Not Reported	Not Reported	Not Reported	Pearson's correlation, mutual Information and Kullback–Liebler methods. greatest Arc strength: 1.0441. The variable Perimeter has a mean of	Not Reported

									33016.174 and deviation of 1260.157, probability 63.41%	
van Egdom et al., 2020	General Linear Model (GLM), (SVM), (ANN), DL	Not Reported	Not Reported	Not Reported	Not Reported	Not Reported	Not Reported	Not Reported	Not Reported	Not Reported
Albers et al., 2021	LR + mixed-effects model	Not reported	Not reported	Not reported	Not reported	Not reported	Not reported	Not reported	p values	Not reported
Bagshaw et al., 2021	Decision making template (Web-based decision aid)	Not reported	Not reported	Not reported	Not reported	Not reported	Not reported	Not reported	value function and preference thresholds	Not reported
Charoenkwan et al., 2021	1) RF(iPMI-Power), 2) SVM, 3) DT, 4) XGB, 5) kNN, 6) iPMI-Econ, 7) MLP, 8) LR, 9) NB	1) 0.905 (95% CI, 0.852-0.958), 2) 0.761 (95% CI, 0.683–0.839), 3) 0.751 (95% CI, 0.672–0.830), 4) 0.873 (95% CI, 0.812–0.934), 5) 0.725 (95% CI, 0.644–0.806), 6) 0.758 (95% CI, 0.680–0.836), 7) 0.843 (95% CI,	1) 0.6, 2) 0.6, 3) 0.6, 4) 0.567, 5) 0.567, 6) 0.267, 7) 0.667, 8) 0.833, 9) 0.867	1) 0.953, 2) 0.872, 3) 0.895, 4) 0.965, 5) 0.884, 6) 0.914, 7) 0.895, 8) 0.767, 9) 0.593	1) 0.862, 2) 0.802, 3) 0.819, 4) 0.862, 5) 0.802, 6) 0.756, 7) 0.836, 8) 0.784, 9) 0.664	Not reported	Not reported	Not reported	1) MCC: 0.54	Not reported

		0.777–0.909), 8) 0.869 (95% CI, 0.808–0.930), 9) 0.844 (95% CI, 0.778–0.910)								
Agochukwu-Mmonu et al., 2022	Gradient Boosting Decision Trees (GBDT)	12-mo EPIC-26 ≥73: 0.82 (baseline), 0.91 (dynamic); 24-mo: 0.81 to 0.94; Erection quality 12-mo: 0.80 to 0.89; 24-mo: 0.81 to 0.92	Not reported	Not reported	Not reported	Not reported	12 m: 24; 24 m: 26	12 m: 20; 24 m: 21	Not reported	Good calibration for EPIC-26 at 12/24 months; underprediction for erection quality at 24 months; visualized with calibration plots
Chan et al., 2022	LR	Not reported	Not reported	Not reported	Not reported	Not reported	Not reported	Not reported	p value	Not reported
Chao et al., 2022	1) LR. 2) GBM	1) LR: 0.891 (95% CI, 0.821–0.960), 2)GBM : 0.942 (95% CI, 0.914–0.969)	1) LR: 88.9%, 2)GBM: 86.8%	1) LR: 76.7%, 2) GBM: 86.7%	Not reported	Not reported	Not reported	Not reported	Not reported	1) LR: Calibration curve was good prediction value, The p-value of the Hosmer-Lemeshow goodness-of-fit test was 0.4, which was >0.05, whereas the C-

										index was 0.891.
Gentile et al, 2022	Neural Network (deep learning)	Not Reported	0.8	0.68	Not Reported	Not Reported	Not Reported	Not Reported	Not Reported	Not Reported
Sun et al., 2022	Stacking-integrated ML algorithm that combined multiple base models, including RF, SGB, TreeBag, XGBoost, MonMLP, SVMRadial, KNN, GaussPrRadial, RgeLogistic, SLDA, LMT.	0.877	0.818	0.819	0.819	0.368	Not Reported	Not Reported	Not Reported	Not Reported
Deng et al., 2023	Random Forest	"RF: Nomogram model: 0.975 Post-LEEP HPV: 0.820 Post-LEEP TCT: 0.894	Not Reported	Not Reported	Not Reported	Not Reported	Not Reported	Not Reported	(C-index) of the nomogram model for predicting residual lesions was 0.975 (0.962–0.988).	Calibration curve demonstrated good consistency of the model , Hosmer-Lemeshow test.

		Gland involvement: 0.773"							DECISION CURVE ANALYSIS. Showed that when the high-risk threshold was set to >0.18, using the nomogram model provided more benefits compared to no treatment or treating all patients.	
Hariprasad et al., 2024	GBM XGBoost RF SVM MLP KNN LR	GBM: 0.996 XGBoost: 0.996 NB: 0.827 Adapoost: 0.992 DT: 0.955 LightGBM: 0.987 RF: 0.997 SVM RBF: 0.986 SVM linear:	GBM: 0.984 XGBoost: 0.986 NB: 0.575 Adapoost: 0.978 DT: 0.960 LightGBM: 0.970 RF: 0.984 SVM RBF: 0.967	"GradientB: 0.988 XGBoost: 0.985 Naive Bayes: 0.835 Ada: 0.977 DT: 0.947 LightGBM: 0.954 Random Forest: 0.989	GradientB: 0.986 XGBoost: 0.986 Naive Bayes: 0.724 Ada: 0.978 DT: 0.953 LightGBM: 0.961 Random Forest: 0.987 SVM RBF:	Gradient Boosting, XGBoost, Naive Bayes, Ada Boost, Decision Tree, LightGBM, Random Forest, SVM using Radial Basis Function(RBF) kernel, SVM	GradientB: 0.986 XGBoost: 0.985 Naive Bayes: 0.675 Ada: 0.977 DT: 0.954 LightGBM: 0.962 Random Forest: 0.987 SVM RBF:	Not Reported	Not Reported	Not Reported

		0.852 SVM poly: 0.843 MLP: 0.976 KNN: 0.956 LR: 0.836	SVM linear: 0.697 SVM poly: 0.686 MLP: 0.918 KNN: 0.989 LR: 0.680"	SVM RBF: 0.891 SVM linear: 0.818 SVM poly: 0.822 MLP: 0.900 KNN: 0.831 LogisticR: 0.794"	0.923 SVM linear: 0.763 SVM poly: 0.749 MLP: 0.906 KNN: 0.891 LogisticR: 0.743	Linear, SVM Polynomial, Multi-Layer Perceptron (MLP), K-Nearest Neighbors(KNN) and Logistic Regression.	0.927 SVM linear: 0.747 SVM poly: 0.731 MLP: 0.907 KNN: 0.902 LogisticR: 0.726			
Hasannejadasl et al., 2023	logistic regression algorithm coupled with Recursive Feature Elimination (RFE).	0.84 for 1 year, 0.81 for 2 years post-diagnosis	8.1%, 87.4%	66.2%, 62.1%	75.3%, 73.7%	Not Reported	Not Reported	Not Reported	Coefficients and FDR corrected q-value	calibration plots and the estimated intercept: 1-year intercept is 0.05485955 and the 2-year is 0.1988651
Lei et al., 2023	DL basedvTopological Modulated Network. Lasso Regression, kNN	Not Reported	Range. 0.68-0.9	Not Reported	L NVB: 1. 85.8, 2. 86.9, 3. 87.4<br><br>R NVB: 1. 85.7, 2. 86.9, 3. 87.4	Not Reported	Not Reported	Not Reported	The Dice similarity coefficient (DSC) and The 95th percentile Hausdorff distance (HD95) are (left NVB) 0.81 ± 0.10, 1.49 ± 0.88 mm,	Not Reported

									and (right NVB) 0.80 ± 0.15, 1.54 ± 1.22 mm, respectively. Others :MSD (mm) CMD (mm) VD (cc)	
Sibert et al., 2023	Lasso Regression	Not Reported	Not Reported	Not Reported	Not Reported	Not Reported	internal validation (sexual fxn model): 21.44<br>external validation (sexual fxn model): 21.71<br><br>internal validation (incontinence model): 25.40<br>external validation (incontinence model): 26.03	Not Reported	Coefficient: R2 internal validation (incontinence model): 0.12<br>R2 external validation (incontinence model): 0.10<br><br>R2 internal validation (sexual fxn model): 0.23<br>R2 external validation (sexual fxn model): 0.22	(QQ plots and fitted values vs. residuals). Calibration-in-the-large was assessed by comparing the mean observed to the mean expected scores, showed good results. Sexual function: both mean predicted and mean observed sexual function scores were

										26.4.Incontinence: the mean predicted incontinence score was 74.0 compared to an observed mean of 73.9. Calibration-in-the-large analysis for the external validation data set was excellent (for incontinence: mean predicted score of 73.7 vs. a mean observed score of 73.4; for sexual: mean predicted score of 26.9 vs. a mean observed score of 26.5).
Xu et al., 2023	Logistic Regression with elastic net,	changes in physical well-being (chest	Not Reported	Not Reported	Not Reported	Not Reported	Not Reported	Not Reported	Not Reported	Not Reported

	XGBoost, and Neural Network	and upper body) (worsened: AUC range 0.69 (95% CI, 0.62–0.76)–0.70 (95% CI, 0.63–0.77); improved: AUC range 0.81 (95% CI, 0.75-0.86)–0.82) (95% CI, 0.76-0.87), sexual well-being (worsened: AUC range 0.76 (95% CI, 0.70–0.82)–0.77 (95% CI, 0.70–0.83); improved: AUC range 0.74 (95% CI, 0.67–0.81)–0.76 (95% CI, 0.70–0.83), and psychosocial well-being (worsened:								

		AUC range 0.64 (95% CI, 0.55–0.72)–0.66 (95% CI, 0.58–0.74); improved: AUC range 0.66 (95% CI, 0.58–0.73)–0.66 (95% CI, 0.59–0.74)								
Balagopal et al., 2024	SNet-MA (U-Net with squeeze-and-excite blocks, modality attention, muscle-bone loss, and modality drop-out)	Not reported	Not reported	Not reported	DSC = 62.2%	Not reported	Not reported	Not reported	DSC 61.71 ± 7.7 %, ASD 2.5 ± .87 mm, and HD95 7.0 ± 2.3 mm.	Not reported
Chauhan et al., 2024	1) adaboost, 2)kNN, 3) nb, 4)SVM,, 5) xgBoost, 6) DT	1) 0.82, 2) 0.86, 3) 0.61, 4) 0.77, 5) 0.91, 6) 0.795	Not reported	Not reported	Not reported	Not reported	Not reported	Not reported	Not reported	Not reported
Devi et al., 2024	<u>Classification Models:</u> NB LR	LR: 0.97 SGD Classifier: 0.96 Naive bayes:	LR: 0.95 SGD Classifier: 0.94 Naive bayes:	LR: 0.95 SGD Classifier: 0.94 Naive bayes:	LR: 94.8% SGD Classifier: 93.9% Naive bayes:	LR: 0.95 SGD Classifier: 0.94 Naive bayes:	Not Reported	Not Reported	Not Reported	Not Reported

	Ensemble Models: RF, Bagging, Soft Voting, Weighted Averaging DL Models: MLP, NN, Long Short-Term Memory LSTM	0.95 Random Forest: 0.98 KNN:0.98 Ensemble-soft voting: 0.98 Ensemble-stacking: 0.97 Ensemble-bagging: 0.99 Ensemble-weighted averaging: 0.98 Neural Network: 0.99 LSTM: 0.97 MLP: 0.99	0.95 Random Forest: 0.98 KNN:0.97 Ensemble-soft voting: 0.95 Ensemble-stacking: 0.95 Ensemble-bagging: 0.98 Ensemble-weighted averaging: 0.94 Neural Network: 0.97 LSTM: 0.96 MLP: 0.98	0.95 Random Forest: 0.99 KNN:0.97 Ensemble-soft voting: 0.95 Ensemble-stacking: 0.95 Ensemble-bagging: 0.99 Ensemble-weighted averaging: 0.94 Neural Network: 0.97 LSTM: 0.96 MLP: 0.99	94.8 Random Forest: 98.5% KNN:98.3% Ensemble-soft voting: 95% Ensemble-stacking: 95.3% Ensemble-bagging: 98.5% Ensemble-weighted averaging: 94.2% Neural Network: 98.5% LSTM: 95.7% MLP: 98.5%	0.95 Random Forest: 0.98 KNN:0.97 Ensemble-soft voting: 0.95 Ensemble-stacking: 0.95 Ensemble-bagging: 0.98 Ensemble-weighted averaging: 0.94 Neural Network: 0.97 LSTM: 0.96 MLP: 0.99				
Hanai et al., 2024	Generative AI (GPT)	Not Reported	Not Reported	Not Reported	Not Reported	Not Reported	Not Reported	Not Reported	mean similarity score between Bot1 and Bot2 responses was 0.93 (ranging from 0.77 to 0.98). Sentiment analysis showed a slightly	Not Reported

									positive polarity (Bot1 mean=0.18 (SD=0.12), Bot2 mean=0.19 (SD=0.15)).	
Saikali et al., 2025	Neural Network (ANN)	0.74 for potency<br>0.68 for continence<br><br>For potency, the AUC values for the comparison models were XGB=0.71 and SVM=0.74. For continence, the AUC values were<br>XGB=0.62, and SVM=0.43.	Not Reported	Not Reported	Not Reported	Not Reported	Not Reported	Not Reported	Not Reported	ANN model calibration was assessed by comparing it to a fraction of positives curve and displaying the Q-Q plot curve, that show a predicted R2 of 0.94 and<br>0.82 for the likelihood of potency and continence at 12 months, respectively.

Supplementary Table S2. Median performance metrics scores among different models.

**AUC**

	**Boosting**	**Neural Network**	**Tree-Based RF**	**KNN**	**Naïve Bayes**	**Regression**	**SVM**
Minimum	0.77	0.68	0.91	0.73	0.61	0.60	0.69
25% Percentile	0.845	0.72	0.93	0.80	0.66	0.66	0.74
Median	0.94	0.79	0.98	0.96	0.83	0.83	0.77
75% Percentile	0.99	0.89	0.99	0.98	0.92	0.89	0.85
Maximum	0.99	0.99	1.00	0.98	0.95	0.97	0.90
Range	0.22	0.31	0.09	0.25	0.34	0.37	0.21
Mean	0.91	0.80	0.97	0.90	0.81	0.79	0.79
Std. Deviation	0.08	0.11	0.04	0.11	0.14	0.13	0.07
Std. Error of Mean	0.03	0.05	0.02	0.05	0.07	0.05	0.03
Lower 95% CI of mean	0.88	0.69	0.91	0.77	0.58	0.69	0.73
Upper 95% CI of mean	0.95	0.92	1.03	1.04	1.03	0.90	0.86
**Sensitivity**							
Minimum	0.87	0.80	0.60	0.57	0.57	0.68	0.60
25% Percentile	0.89	0.80	0.60	0.57	0.57	0.76	0.60
Median	0.96	0.89	0.98	0.97	0.87	0.87	0.65
75% Percentile	0.99	0.97	0.98	0.99	0.95	0.92	0.70
Maximum	0.99	0.97	0.98	0.99	0.95	0.95	0.70
Range	0.12	0.17	0.38	0.42	0.38	0.27	0.10
Mean	0.94	0.89	0.85	0.84	0.80	0.84	0.65
Std. Deviation	0.05	0.12	0.22	0.24	0.20	0.10	0.07
Std. Error of Mean	0.03	0.09	0.13	0.14	0.12	0.05	0.05
Lower 95% CI of mean	0.86	-0.20	0.31	0.25	0.30	0.72	0.01

Up-per 95% CI of mean	1.03	1.97	1.40	1.43	1.29	0.97	1.29
Specificity							
Minimum	0.94	0.97	0.95	0.83	0.59	0.66	0.82
25% Percentile	0.94	0.97	0.95	0.83	0.59	0.69	0.82
Median	0.97	0.97	0.99	0.88	0.84	0.78	0.85
75% Percentile	0.99	0.97	0.99	0.97	0.95	0.91	0.87
Maximum	0.99	0.97	0.99	0.97	0.95	0.95	0.87
Range	0.05	0.00	0.04	0.14	0.36	0.29	0.05
Mean	0.97	0.97	0.98	0.89	0.79	0.79	0.85
Std. Deviation	0.03	0.00	0.02	0.07	0.18	0.12	0.04
Std. Error of Mean	0.01	0.00	0.01	0.04	0.11	0.06	0.03
Lower 95% CI of mean	0.90		0.92	0.72	0.34	0.60	0.53
Up-per 95% CI of mean	1.03		1.03	1.07	1.25	0.98	1.16
F1 Score							
Minimum	0.94	0.97	0.98	0.9	0.68	0.73	0.73
25% Percentile	0.94	0.97	0.98	0.9	0.68	0.73	0.73
Median	0.965	0.97	0.985	0.935	0.815	0.84	0.73
75% Percentile	0.99	0.97	0.99	0.97	0.95	0.95	0.73
Maximum	0.99	0.97	0.99	0.97	0.95	0.95	0.73
Range	0.05	0	0.01	0.07	0.27	0.22	0
Mean	0.965	0.97	0.985	0.935	0.815	0.84	0.73
Std. Deviation	0.03536	0	0.007071	0.0495	0.1909	0.1556	0
Std. Error of Mean	0.025	0	0.005	0.035	0.135	0.11	0
Lower 95% CI of mean	0.6473		0.9215	0.4903	-0.9003	-0.5577	

Up-<br>per 95% CI of mean	1.283		1.049	1.38	2.53	2.238	
