# Supplementary material for: Artificial Intelligence and Machine Learning in Sexual Health and Dysfunction Across the Cancer Care Continuum: A Systematic Review
Source: Cancers (Basel). 2025 Sep 16;17(18):3025. doi: 10.3390/cancers17183025 (PMC12468962; doi:10.3390/cancers17183025)

Supplementary File S1; Research Strategy of Databases:

PubMed

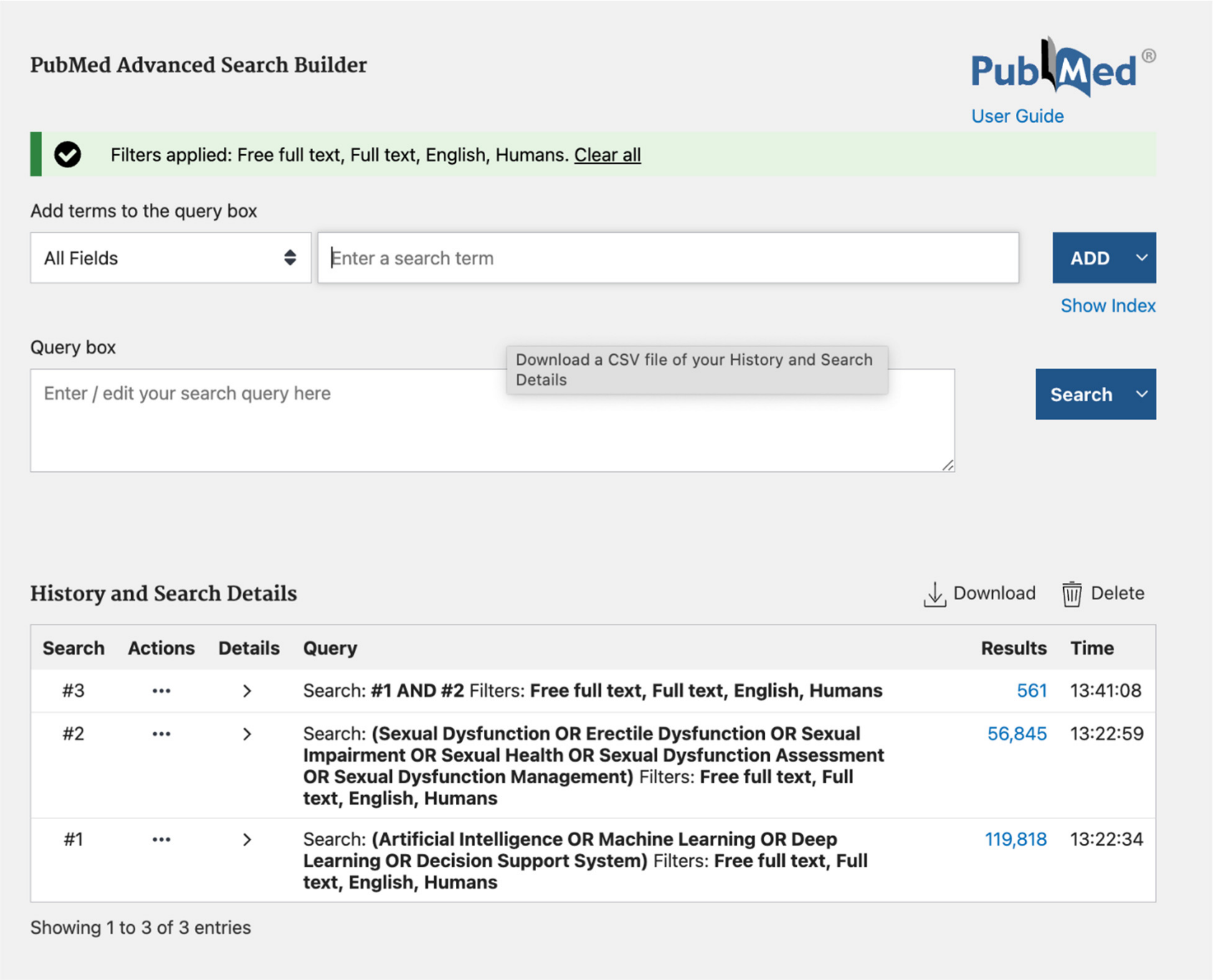

RESULTS BY YEAR

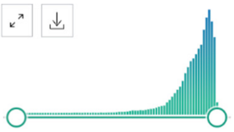

PUBLICATION DATE

- ○ 1 year
- ○ 5 years
- ○ 10 years
- ○ Custom Range

TEXT AVAILABILITY

- ☐ Abstract
- ☑ Free full text
- ☑ Full text

ARTICLE ATTRIBUTE

- ☐ Associated data

ARTICLE TYPE

- ☐ Books and Documents
- ☐ Clinical Trial
- ☐ Meta-Analysis
- ☐ Randomized Controlled Trial
- ☐ Review
- ☐ Systematic Review

See all article type filters

Additional filters (2) –

ARTICLE LANGUAGE ⓘ

- ☑ English
- ☐ Spanish

See all article language filters

SPECIES ⓘ

- ☑ Humans
- ☐ Other Animals

SEX ⓘ

Filters applied: Free full text, Full text, English, Humans. Clear all

☐ 1 Cite Share

Autonomic Dysfunction in Parkinson's Disease.
Pfeiffer RF.
Neurotherapeutics. 2020 Oct;17(4):1464-1479. doi: 10.1007/s13311-020-00897-4.
PMID: 32789741 Free PMC article. Review.
Recognition of the importance of nonmotor **dysfunction** as a component of Parkinson's **disease** has exploded over the past three decades. ...Urinary **dysfunction** can entail either too frequent voiding or difficulty voiding. **Sexual dysfunction** is freq ...

☐ 2 Cite Share

International Society for the Study of Women's **Sexual Health** Clinical Practice Guideline for the Use of Systemic Testosterone for Hypoactive **Sexual** Desire Disorder in Women.
Parish SJ, Simon JA, Davis SR, Giraldi A, Goldstein I, Goldstein SW, Kim NN, Kingsberg SA, Morgentaler A, Nappi RE, Park K, Stuenkel CA, Traish AM, Vignozzi L.
J Sex Med. 2021 May;18(5):849-867. doi: 10.1016/j.jsxm.2020.10.009. Epub 2021 Apr 1.
PMID: 33814355 Free article. Review.
RESULTS: Although the Global Position Statement endorses testosterone therapy for only postmenopausal women, limited data also **support** the use in late reproductive age premenopausal women, consistent with the International Society for the Study of Women's **Sexual** ...

☐ 3 Cite Share

Hypoactive **Sexual** Desire Disorder in Women: Physiology, **Assessment**, Diagnosis, and Treatment.
Pettigrew JA, Novick AM.
J Midwifery Womens Health. 2021 Nov;66(6):740-748. doi: 10.1111/jmwh.13283. Epub 2021 Sep 12.
PMID: 34510696 Free PMC article. Review.
Nearly half of women in the United States report problems with **sexual** function. Many **health** care providers do not ask about **sexual** concerns during routine clinical encounters because of personal discomfort, lack of familiarity with treatment, or the belief th ...

☐ 4 Cite Share

Premature ejaculation: A clinical review for the general physician.
Chung E, Gilbert B, Perera M, Roberts MJ.
Aust Fam Physician. 2015 Oct;44(10):737-43.
PMID: 26484490 Free article. Review.
It can potentially lead to **psychological** distress, diminished self- esteem, anxiety, **erectile dysfunction**, reduced libido and poor interpersonal relationships. Most men feel reluctant to discuss premature ejaculation with their general practitioner despite it ...

☐ 5 Cite Share

Adverse **Sexual** Effects of Treatment with Finasteride or Dutasteride for Male Androgenetic Alopecia: A Systematic Review and Meta-analysis.
Lee S, Lee YB, Choe SJ, Lee WS.
Acta Derm Venereol. 2019 Jan 1;99(1):12-17. doi: 10.2340/00015555-3035.
PMID: 30206635 Free article.
Use of 5alpha-reductase inhibitors carried a 1.57-fold risk of **sexual dysfunction** (95% confidence interval (95% CI) 1.19-2.08). The relative risk was 1.66 (95% CI 1.20-2.30) for finasteride and 1.37 (95% CI 0.81-2.32) for dutasteride. ...It is important that physici ...

☐ 6 Cite Share

Maca (L. meyenii) for improving **sexual** function: a systematic review.
Shin BC, Lee MS, Yang EJ, Lim HS, Ernst E.
BMC Complement Altern Med. 2010 Aug 6;10:44. doi: 10.1186/1472-6882-10-44.
PMID: 20691074 Free PMC article. Review.
Preparations from maca root have been reported to improve **sexual** function. The aim of this review

EMBASE Database search

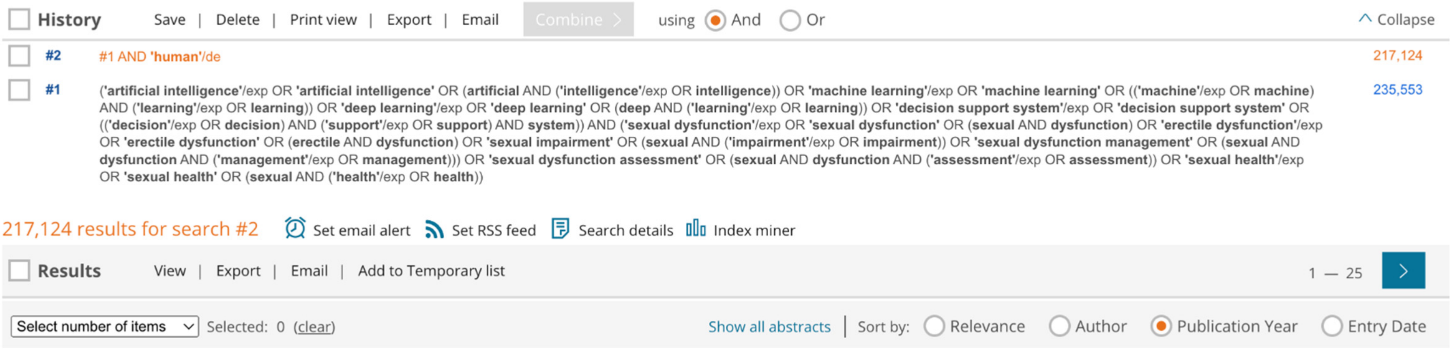

Web of Science

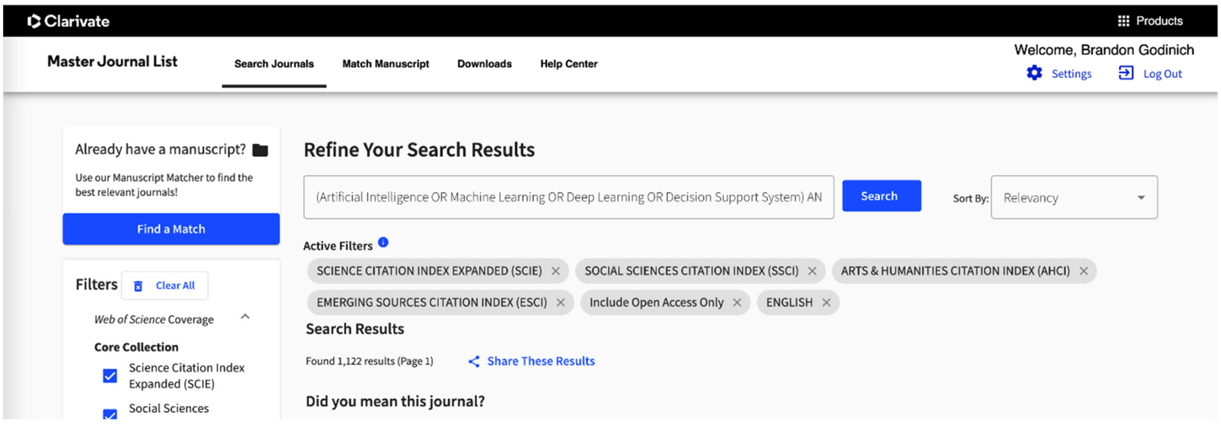

Supplement: Supplementary file 1 [file cancers-17-03025-s001.zip › Supplementary file S1.pdf]
